# Phenotypic diversity in qualitative and quantitative traits for selection of high yield potential field pea genotypes

**DOI:** 10.1038/s41598-024-69448-7

**Published:** 2024-08-09

**Authors:** Mohammad Golam Azam, Umakanta Sarker, Mohammad Amir Hossain, A. K. M. Mahabubul Alam, Mohammad Rafiqul Islam, Nazmul Hossain, Saud Alamri

**Affiliations:** 1 Pulses Research Centre, Bangladesh Agricultural Research Institute, Ishurdi, Pabna, 6620 Bangladesh; 2 Department of Genetics and Plant Breeding, Faculty of Agriculture, Bangabandhu Sheikh Mujibur Rahman Agricultural University, Gazipur, 1706 Bangladesh; 3 Department of Genetics and Plant Breeding, Faculty of Agriculture, Bangladesh Agricultural University, Mymensingh, 2202 Bangladesh; 4 Pulses Research Sub-Station, Bangladesh Agricultural Research Institute, Gazipur, 1701 Bangladesh; 5 Agronomy Division, Regional Agricultural Research Station, Bangladesh Agricultural Research Institute, Pabna, 6620 Bangladesh; 6 Department of Agronomy, Iowa State University, Ames, IA 50010 USA; 7 Department of Botany and Microbiology, College of Science, King Saud University, Riyadh, Saudi Arabia

**Keywords:** Plant breeding, Agricultural genetics

## Abstract

Field pea (*Pisum sativum* L.) needs improvement to increase productivity due to its high price and demand. However, the incidence of powdery mildew (PM) disease limits its production. This study aimed to analyze the diversity of qualitative and quantitative traits against powdery mildew resistance by utilizing cluster and principal component analysis to explore PM resistance high-yield potential field peas. Shannon–Weaver's diversity index (Hʹ) displayed high intra-genotype diversity for quantitative and qualitative aspects. Heterogeneity was identified for resistance against powdery mildew infections. Eighty-five genotypes were divided into five groups using Mohalanobis generalized distance (D^2^) statistics. The highest inter-cluster D^2^ value was observed between clusters 2 and 3 (11.89) while the lowest value was found between clusters 3 and 4 (2.06). Most of the genotypes had noticeable differences, so these could be employed in a crossing scheme. Twelve genotypes were extremely resistant, 29 genotypes were resistant, 25 genotypes were moderately resistant, 18 genotypes were fairly susceptible, and 1 genotype was susceptible to powdery mildew disease. Among 29 resistant genotypes, BFP77, BFP74, BFP63, BFP62, BFP43, and BFP80 were high yielders and, could be used directly and/or transferred through hybridization to high-yielding disease-susceptible genotypes. Among the 25 moderately resistant genotypes, BFP78, BFP45, BFP79, and BFP48 were found to be high yielders. In principal component analysis (PCA), the first four PCs with Eigen values > 1 accounted for 88.4% variability for quantitative traits. Clustering sorted genotypes into five groups, where groups 1 to 5 assembled 37, 28, 1, 8, and 11 genotypes, respectively. Genotypes of cluster 4 were identified as high yielders with its attributes. Pearson correlation significantly and positively correlated across all traits except for PM. This variation suggested that there is a mechanism to select promising genotypes for field pea breeding. Considering all features, BFP78, BFP77, BFP74, BFP63, BFP62, BFP45, BFP79, and BFP80 could be preferred as high yielders and PM resistance owing to longer pod lengths, seeds per pod and pods per plant.

## Introduction

Field pea (*Pisum sativum* L.) is one of the oldest crops in cultivation and the third-largest producer of legumes worldwide^1^. It has chromosome number 2n = 14 and is a self-pollinated diploid. In tropical, subtropical, and temperate parts of the world, field pea is a significant cool-season multifunctional crop that is widely farmed for grain and green pods^2,3^. It is recognized as one of the highly productive, nutritionally dense cool-season legume crops, which has great promise for reducing protein deficiency in the resource-constrained society^4^. With a yield of over 16.20 metric tons and a global cultivation area of over 8.14 million hectares, it meets consumer demand for food, feed, and fodder^5^. Due to numerous widespread biotic and abiotic stresses, of which powdery mildew, rust, and high temperatures are the main concerns^6,7^ the productivity of dry peas in Bangladesh is very low and fluctuates between 1120 and 1338 kg/ha. According to Azam et al.^8^ and USDA^9^, the crop is mainly grown for human consumption, animal feed, and as a source of income for farmers. For the human diet, it is a significant source of proteins and other nutrients^10^. Additionally, the crop is highly valuable economically since it can grow in low-rainfall areas better than other pulses like faba beans and lentils^11^. However, the nation imports pulse waste at a cost of around 645^12^. Parihar et al.^13^ noted that pea seeds have an average amount of iron, selenium, zinc, and molybdenum of around 97, 42, 41, and 12 ppm, respectively. They are also considered to have 21–33% protein and 56–74% carbohydrates. Field peas are high in lysine and tryptophan amino acids and low in cysteine and methionine amino acids^14,15^ these are rich sources of protein (21–25%). Being a leguminous vegetable, it occupies a prominent position among vegetables due to its high nutritional value, particularly proteins and a variety of other health-improving ingredients like carbohydrates, vitamin A, vitamin C, calcium, phosphorus, and essential amino acids, particularly lysine^16^. As a result, it plays a crucial role in maintaining nutritional security for those who lack access to resources in impoverished countries. By fostering a symbiotic relationship between nitrogen-fixing bacteria and plant root nodules, they can increase soil fertility^17^. In several locations in Bangladesh, it is cultivated primarily as a relay crop alongside transplant Aman rice. After harvesting the pods, the plants are used as either feed or organic soil amendments, and the green pods are sold as food.

It is a member of the Fabaceae family, the largest family of flowering plants with 1200 species and 450 genera^18^. The cotyledons of pea grains come in a variety of colors, including yellow, green, and orange. These grains are used in dal, stew, chhola, vegetables, snacks, soup, chat, and flour, whereas entire seeds are mostly used as animal feed^19,20^. It is also helpful in food processing because it can be transformed into a variety of products, including biscuits, pasta, salad, noodles, cookies, and bread^21^. This makes it extremely relevant economically. Smallholder farmers in rural areas of several developing nations, like Bangladesh, find it to be quite alluring^8^. Field pea serves as a unique food supply during the lean season with minimal to no value addition, as well as function as feed for animals, green vegetables for humans, and an income source for both men and women^7^. Field pea seeds are used as a raw material in a variety of industries to make goods with added value including *Bombay Chanachur*, *Ruchi,* and *PRAN Dal Vaja*.

It is extensively grown in a variety of nations throughout the globe^22^. Since 2010, its global cultivable area has expanded from 6.58 to 8.09 million hectares, and its output has increased from 10.44 to 16.21 metric tons. The main producers of peas are Canada, Russia, China, India, and the United States^23^ nevertheless; the United States produces the most peas overall 39.33%, followed by Europe (36.98%) and Asia (18.09%). The crop's average productivity is currently 2.0 t/ha worldwide, up about 36% over the past ten years (2007–2017), but it has the potential to produce up to 5.0 t/ha in many nations, including the Netherlands, Denmark, Belgium, Germany, and Finland, where harvests range from 3.45 to 5.01 t/ha^24^. However, nations with productivity levels of less than 2.00 t/ha include Australia, China, India, and Myanmar^25^.

Peas have poor output and productivity due to their susceptibility to many fungal infections and parasites^26^. *Erysiphe polygoni* a filamentous fungus responsible for 25–70% of crop losses in pea production causes pea powdery mildew, an airborne fungal disease^27,28^. Nisar et al.^29^ found that powdery mildew disease reduced the production potential of field pea germplasm grown in various regions of the globe by 86%. In the mid-altitudes and with moderate intensity, powdery mildew disease has been observed to reduce field pea output by 20–30%. When the days are warm and dry and the nights are icy enough for dew to accumulate, powdery mildew is a bothersome illness. Generally, the price of field peas is a little bit high in Bangladesh though it is affordable to consumers. However, sometimes yield loss by PM severely affects farmers' incomes, as the government can’t support farmers with crop insurance. Furthermore, the loss of yield of field peas reduces the market supply, and prices of field peas hike at the consumer level. To prevent the illness, farmers often employ chemical agents, which might pollute the environment^30^. Eklund et al.^31^ showed that spore discharge may potentially affect farm employees' respiration and allergy responses. Due to the high expense of fungicides, adverse effects on the environment, social and health issues, and other factors, it is preferable to search out other alternative ways of disease management. Crop breeding should prioritize genetically based resistance^32^. To enhance and maintain the output and productivity of field peas for small-scale farmers, it is necessary to produce high-yielding and powdery mildew resistant cultivars^33^. The development of superior crop varieties depends heavily on their existing genetic variation and proper selection against yield-attributed traits^34–36^. The degree of genetic diversity and variability^37,38^ and the degree of inheritance of required traits determine the achievement of up gradation in breeding programs^39^. Approaches using multivariate analysis may be useful in identifying genetic diversity and classifying germplasm^40^. Principal component analysis (PCA), one of these methods, uses statistics to group a large number of variables into fundamental, uncorrelated components. PCA may be used to determine germplasm characterization traits, show individual differences and relationships, and evaluate each individual's contribution to overall variance^41^. In addition, germplasm may be categorized and organized using hierarchical cluster analysis^42–44^.

Selecting superior parents for hybridization will be aided by an evaluation of the kind and degree of variety^45^. Principal component analysis is a method used to decrease the size of large datasets, improve interpretability, and limit information loss^46^. Designing breeding programs for novel objectives may be aided by estimates of the variety of these characteristics, cluster analysis, and PCA^47^. For the preservation, breeding strategies, development, and commercialization of new varieties^46,47^, high-yielding phenotypic variability provided by various morphological and agronomical characteristics, such as seed, leaf, plant, and fruit-related characteristics, is crucial to understand^48–51^.

The economic yield, a very complicated characteristic of crops, is influenced by local climate and genetic factors. The crop also experiences many biotic and abiotic stressors and exhibits poor response to inputs^52^. To increase the genetic variety and revitalize breeding stocks, new germplasm must be introduced. So, reducing the present yield gap and creating high-yielding types depend on productivity and growth crops. Because of their comparatively greater economic significance, early genotypes in particular still have poor productivity, mostly because there aren't many kinds with stable, high-yielding potential and losses from a variety of biotic and abiotic stressors^53^. A comprehensive evaluation of the field pea's qualitative and quantitative traits is however uncommon. To develop crop advancement programs that produce high-yielding progeny, it is thus necessary to investigate genetic variability^54^. The necessity for a varied parent to produce better genotypes in segregating generations was discussed by many researchers^1,55,56^. Yield-related traits have been considered by many researchers in multivariate analysis to categorize and estimate diversity in crops. The researchers have a great interest in developing new varieties with the improvement of qualitative^57–60^ and quantitative traits^61–64^ based on stability^65,66^, variability, and diversity^67–70^. Pea is an important vegetable crop as well as legumes in Bangladesh. In the country, a limited number of low-yielding pea genotypes are being used commercially which are severely infested by powdery mildew (PM) during the growing period resulting in reduced pea yield. Therefore, the current study was carried out to assess the genetic divergence of significant morpho-agronomic traits and to evaluate the performance of various genotypes of field peas to identify desirable high-yielding pea genotypes with tolerance to PM and find out the genotypes for desirable traits that could be used in pea breeding programs to broaden the gene pool of this important vegetable crop.

## Methods

### Plant materials and site

This research employed a total of 85 different genotypes of field peas for characterization and assessment. The Plant Genetic Resources Centre (PGRC), Bangladesh Agricultural Research Institute (BARI), provided seeds for 23 Indigenous types, 7 genotypes from various agro-ecological areas in Bangladesh, 32 exotic types for exploration through Australia, 1 exotic types from Nepal, 5 released varieties from BARI, 1 released variety from BSMRAU, and 14 were collected from the Pulses Research Centre (PRC), BARI, for these experiments. Released variety BFP84 (BARI Motor-2) was used as a check for powdery mildew scoring in studied genotypes. Table 1 gives the complete details of these genotypes. Pea seeds used for the study comply with our institutional, national, and international guidelines and legislation.

**Table 1:** List of 85 studied field pea genotypes with source and their code. BARI: Bangladesh Agricultural Research Institute; BSMRAU: HRC: Oilseed Research Centre; PGRC: Plant Genetic Resources Centre; PRC: Pulses Research Centre; Rel. var.: Released variety.

Code	Genotypes name	Sources	Code	Genotypes name	Sources
BFP01	BARI Motorshuti-1	Rel. var. BARI	BFP44	BD-4162	PGRC, BARI
BFP02	34,223	India	BFP45	BD-4209	PGRC, BARI
BFP03	BD-4185	PGRC, BARI	BFP46	BD-4181	PGRC, BARI
BFP04	BD-7215	PGRC, BARI	BFP47	ATC-1391	Australia
BFP05	PR-MAGHEE	Australia	BFP48	Mammoth Poded	Australia
BFP06	Natore Local-2	Local	BFP49	Asmora	Australia
BFP07	PEA-88	India	BFP50	BATIUS	Australia
BFP08	TM MAGHEE	Australia	BFP51	Helena	Australia
BFP09	TM CHAITY	Australia	BFP52	10	Australia
BFP10	BD-7211	PGRC, BARI	BFP53	A-125	Australia
BFP11	BD-4223	PGRC, BARI	BFP54	Dunwa	Australia
BFP12	BD-9055	PGRC, BARI	BFP54	Dunwa	Australia
BFP13	BD-9059	PGRC, BARI	BFP55	ATC-853	Australia
BFP14	BD-4182	PGRC, BARI	BFP56	WIRAIG	Australia
BFP15	BFDX-15001	PRC, BARI	BFP57	A-35	Australia
BFP16	BD-4153	PGRC, BARI	BFP58	A-159	Australia
BFP17	SE-2010	Australia	BFP59	A-204	Australia
BFP18	BFP-07005	PRC, BARI	BFP60	A-158	Australia
BFP19	BD-4190	PGRC, BARI	BFP61	Greitauaj	Australia
BFP20	BPD-07001	PRC, BARI	BFP62	Poisgnis	Australia
BFP21	BFP-11016	PRC, BARI	BFP63	A-23	Australia
BFP22	Narail	Local	BFP64	ATC-3489	Australia
BFP23	Jhikargacha Local	Local	BFP65	Kaspa	Australia
BFP24	BD-7217	PGRC, BARI	BFP66	ATC-2499	Australia
BFP25	AVRDCE-241	Australia	BFP67	IPSA Garden pea-3	BSMRAU
BFP26	PR-CHAITY	Australia	BFP68	BD-4142	PGRC, BARI
BFP27	BD-4158	PGRC, BARI	BFP69	BFP-22010	Australia
BFP28	BFD-07004	PRC, BARI	BFP70	BFP-22011	Australia
BFP29	BARI Motorshuti-3	Rel. var. BARI	BFP71	BFP-22012	Australia
BFP30	BFPX-91001	PRC, BARI	BFP72	Sikim local	Nepal
BFP31	BD-4228	PGRC, BARI	BFP73	Faridpur local	Local
BFP32	BD-4493	PGRC, BARI	BFP74	BFP-15009-1	PRC, BARI
BFP33	V-34223	Australia	BFP75	BFP-15004-6	PRC, BARI
BFP34	BD-4159	PGRC, BARI	BFP76	BFP-15004-1	PRC, BARI
BFP35	Natore Local-1	Local	BFP77	BFP-15002-2	PRC, BARI
BFP36	Bagha local	Local	BFP78	BFP-15004-8	PRC, BARI
BFP37	BFP-07001	PRC, BARI	BFP79	BFP-15004-5	PRC, BARI
BFP38	BD-4189	PGRC, BARI	BFP80	BFP-15004-3	PRC, BARI
BFP39	BD-4166	PGRC, BARI	BFP81	BD-7047	PGRC, BARI
BFP40	BD-9047	PGRC, BARI	BFP82	BFP-22001	Australia
BFP41	BD-9052	PGRC, BARI	BFP83	BARI Motor-1	Rel. var. BARI
BFP42	LN-CHAAITY	Australia	BFP84	BARI Motor-2	Rel. var. BARI
BFP43	BFP-11015	Local	BFP85	BARI Motor-3	Rel. var. BARI

### Designing experiments and crop management

The field experiment was carried out during the cropping season (November to February) of the years 2022 and 2023 at the field of the Pulse Breeding Division, PRC, BARI, Ishwardi, Pabna, Bangladesh, which is situated at a mean height of 15–19 m above sea level and is located at 24.75°N latitude and 88.5°E longitude. A Randomized Complete Block Design (RCBD) with three replications was used to set up the research. The unit plot for continuous line sowing was 4.0 m by 1 m (two lines). Plant-to-plant spacing was kept at 5–7 cm inside a row. The use of pre-sowing irrigation helped achieve optimal germination. The experimental plots were well-prepared before planting, and we added Farm Yard Manure (FYM). Field pea cultivation was done using the recommended amounts of manure and fertilizer^71^. The seeds were treated with Provex at 2.5 g/kg seed prior to sowing. The experiment was routinely weeded, and irrigation was given as needed. As and when required, other cultural activities were carried out.

### Determination of agro-morphological and qualitative characteristics

Ten competing plants were chosen at random from each entry in each replication, and the observations were averaged by dividing each value by ten. Data were recorded on days to 80% flowering (DF), days to maturity (DM), plant height (PH), pod length (cm) (PL), seeds per pod (SPP), pods per plant (PPP), hundred seed weight (HSW), and yield per plant (YPP). Data on grain yield and yield-related traits were collected on a plant basis. The days to flowering (DF) and days to maturity (DM) were taken when each block reached 80% flowering and 90% physiological pod maturity and calculated from the time required to the date of sowing to the date of 80% flowering and 90% physiological pod maturity, respectively. Plant height (PH), pod length (PL), seeds per pod (SPP), pods per plant (PPP), and yield per plant (YPP) were collected after harvesting from ten randomly selected plants in each plot. Plant height was measured in cm from the base of the plant to the tip of the plant. Pod length was measured from ten randomly selected pods using a scale in cm. The number of seeds in ten randomly selected pods was collected to measure number of seeds per pod. The number of pods in ten randomly selected plants was calculated to measure pods per plant. A hundred dried and cleaned seeds were counted and weighed to measure hundred seed weight (HSW). Ten randomly selected plants were collected and the seeds were separated, dried, and cleaned to measure yield per plant. A set of local descriptors that were produced with the help of several scientific descriptors from the European Union descriptor (UPOV) and IBPGR were used to capture the qualitative characteristics^72,73^.

### Disease data scoring

Early stage, flowering stage, and pod setting stage disease reactions of powdery mildew for various genotypes were documented on a full plot basis 60 days after planting at three times^74^ (Table 2). After seed germination, 10 plants were selected at random from each line and scored for morphological data and powdery mildew. The development of the powdery mildew disease was started by natural infection, and the disease infection was created by spraying a fungicide Benomyl at 2.5 kg/ha at a fixed spray interval of every 7, 14, and 21 days^75^. Using a knapsack sprayer with a 60.6 ml spray volume per 2.4 m^2^ plot, a fungicide was administered. The application of fungicides began as soon as the first detectable disease symptom appeared. The disease scoring process used a scale of 1 to 9 are details presented in Table 2. According to Singh^76^ the disease data collected using the aforementioned scale was translated to a percentage disease index (PDI). Percentage disease index (PDI) was calculated for each genotype using the formula:

**Table 2:** Disease scoring scale used for powdery mildew infection.

Disease scale	Leaf area affected	Response	Remarks
1	0.1–5%	Immune (I)	One or 2 pustules on a few leaves
2	5.1–10%	Highly resistant (HR)	Few pustules on some leaves
3	10.1–17%	Resistant (R)	Few isolated pustules on most of the leaves
4	17.1–25%	Moderately resistant (MR)	Many pustules on most of the leaves
5	25.1–50%	Moderately susceptible (MS)	Many pustules coalescing to each other
6	50.1–75%	Moderately susceptible (MS)	Coalescing pustules on almost the whole plant
7	75.1–90%	Susceptible (S)	Almost uniform powdery growth covering leaves and pods
8	90.1–95%	Highly susceptible (HS)	Uniform powdery growth without any conspicuous pustules on the leaves, pods, and stem
9	95.1–100%	Highly susceptible (HS)	Uniform powdery growth without any conspicuous pustules on the leaves, pods, and stem

$$\text{PDI }(\text{\%}) = \frac{\text{Sum of all ratings}}{\text{Maximum disease grade }\times \text{Total number of observed plants}} \times 100$$

### Statistical analysis

Both quantitative and qualitative characteristics were taken to evaluate the varieties. 11 qualitative and 8 quantitative measurements were combined. Five plants and five pods per plant per accession were used to gather the observations. Each treatment from all the sample data of a trait was averaged to obtain a replication mean. Statistics and biometrics were used to assess the average data of different quantitative traits. The ANOVA, descriptive statistics, and LSD were calculated by using Statistix 8. Microsoft Excel was used to assess the phenotypic diversity for each qualitative feature using the Shannon–Weaver diversity index (Hʹ)^77^. Excel was used to generate the Standardized H' using the following formula^77^:

$$\sum {\left[ {\left( {{\text{pi}}} \right) \times \log \left( {{\text{pi}}} \right)} \right]}$$

where Hʹ—Shannon diversity index, and n—Individuals of a certain kind or species, N- Total number of individuals of a community. According to Eticha et al.^78^, the diversity index was categorized as low (0.10 ≤ H ≤ 0.40), intermediate (0.40 ≤ H ≤ 0.60), or high (H ≥ 0.60).

Using the doe-bioresearch packages, the findings for various agro-morphological parameters were evaluated. The means were separated using the least significant difference at a 5% level of significance. Pooled data from two years (2022 and 2023) were used to perform all analyses. The tools R (v 4.0.5) and R Studio were used to conduct the multivariate analysis^79^. Principal component analysis (PCA) was used to estimate the degree of connection between characteristics, and cluster analysis was used to group genotypes based on traits. Using R's Complex Heatmap package, the two-way hierarchical clustering heatmap was created using the Ward D^2^ and Euclidean distance algorithms. The R packages ggplot2, Factoextra^80^, and FactomineR^81^, were used to create the PCA-biplot. The R program corrplot was used to create the correlation matrix^82^ which was then arranged in hclust. Cluster analysis was used to determine the cluster means and standard deviations for each characteristic. Cluster analysis assessed the cluster mean values, distance values, and Dendrogram; PCA calculated Eigenvalues, variability, cumulative variability, and vector components.

## Results

### Qualitative traits

**Figure 1:**
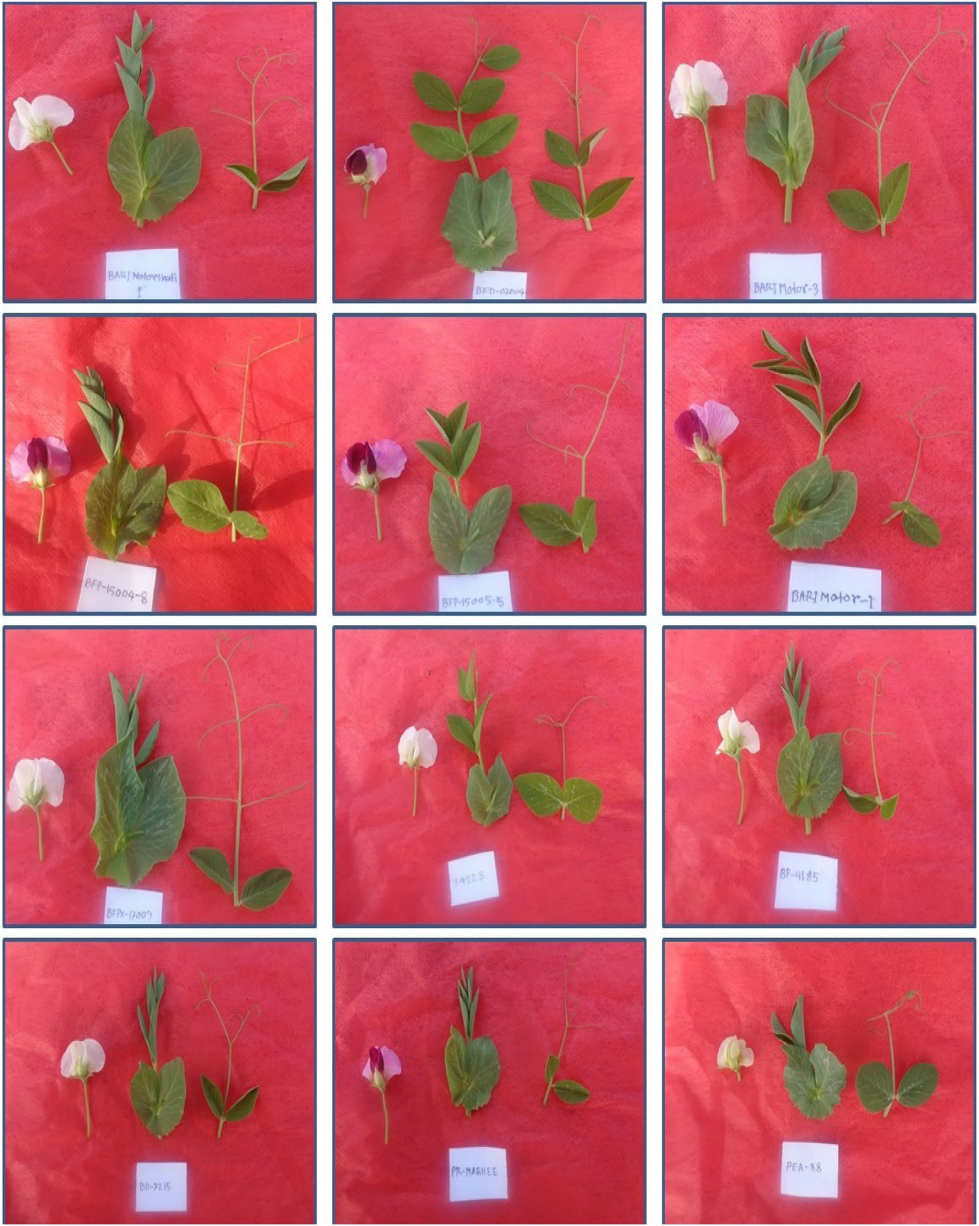

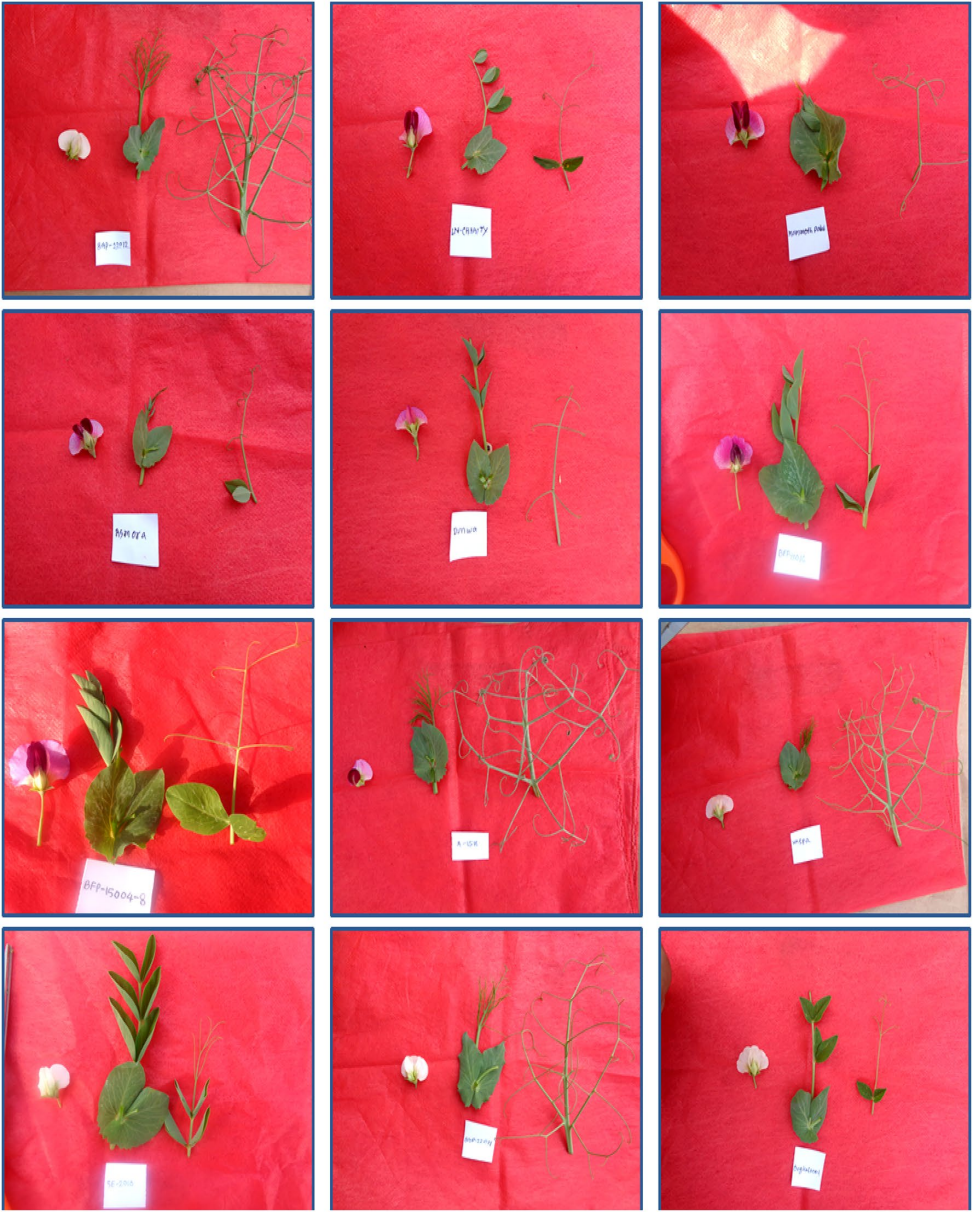
Flower color variation and variability of leaf and tendril structure of field pea genotypes.

**Figure 2:**
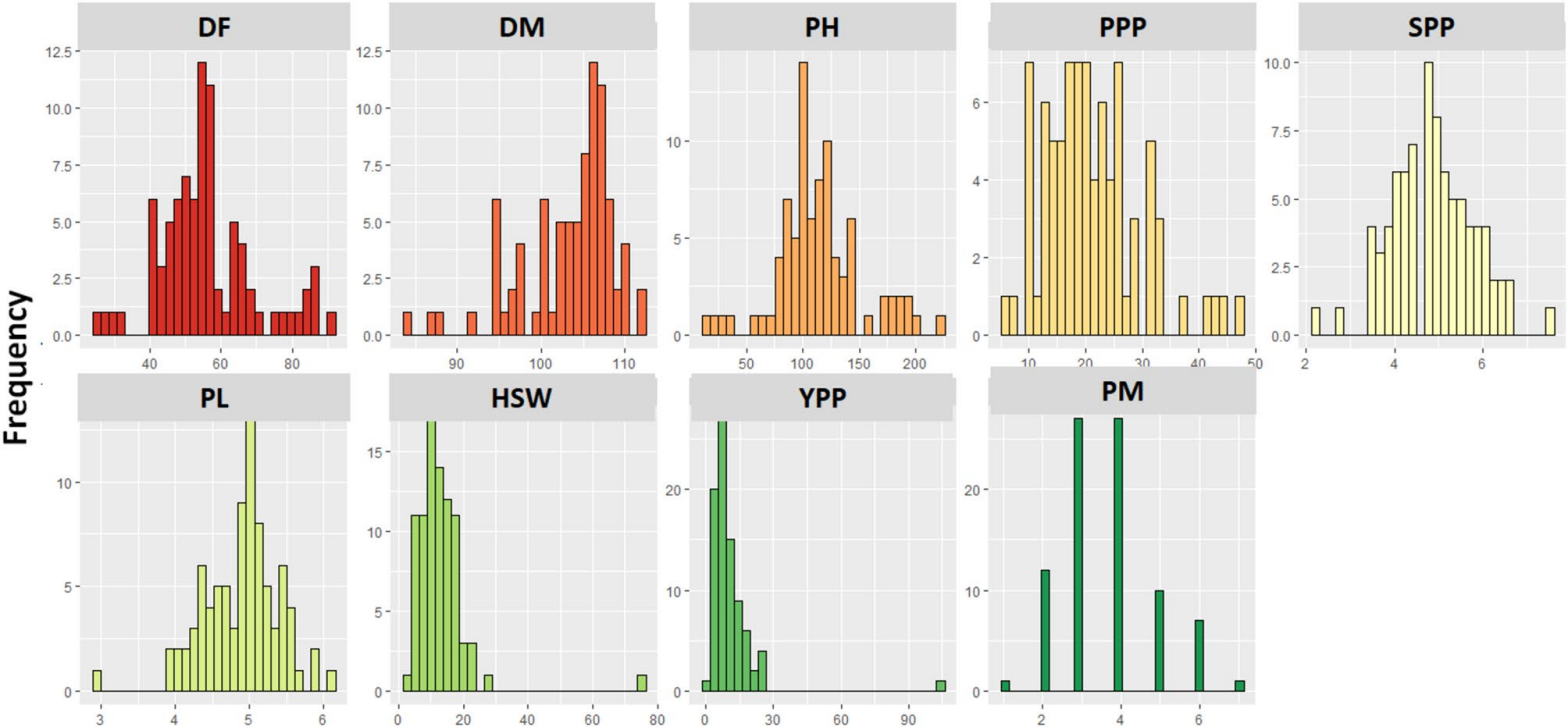
Distribution of 85 field pea genotypes for the eight yields and yield contributing traits.

The qualitative traits of the tested field pea genotypes are presented in Table 3, and the conversion values of the individual traits are exposed in the same Table 3. Flower color variation and variability of leaf and tendril characteristics are shown in Fig. 1. Our results showed significant variation among the traits studied in the field pea genotypes. The leaves were green in 69 genotypes (76.47% of the total), and the remaining 12 genotypes had yellow-green leaves (14.12%). It was found that the color of the tendrils of the collected genotypes was mainly green (56.47%), followed by pale green and purple green tendrils 36.47, and 7.06%, respectively. High leaf sizes were found in medium (43.53%), small, large, very small, and very large (43.53%), 29.41, 16.47, and 3.53%, respectively. Most genotypes of flowers (59 of 85) were creamy white (69.41%). Great diversity in immature pod color was identified. Green, light-green, slight dark green, and slight light green constituted the major types with 56.47, 16.47, 14.12, and 10.59% of the total variation, while the remaining genotype had dark-green colored pods (2.35% of the total). Maximum twining tendrils showed intermediate type (60%) and the lowest found high twining tendrils (16.47%). Two types of growth patterns were recognized in the evaluated genotypes: erect (74.12%) and flat (25.88%). Rough-type pod texture was recorded among the collected field pea genotypes, followed by smooth and tuberculate types. The majority of genotypes had weakly curved pods (72.94%) and a little absent pod curvature (14.12%). Regarding seed size, three types, medium (72.94%), small (20.00%), and large (7.06%)—were recorded from the field pea genotype. The seed colors of the collected genotype are mainly Whitish green (51.76%) and Cream (29.41%). In the case of the seed wrinkle, two types of variation were noted. The frequency distribution of genotypes studied for quantitative traits is shown in Fig. 2. In the histogram, there was much variation found in the evaluated genotypes among the tested traits (Fig. 2).

**Table 3:** Shannon Weaver diversity index (Hʹ), descriptor states, and frequency of qualitative traits.

Traits	Rank	Phenotypic Class	Frequency	Proportion (%)	Hʹ
Leaf color	1	Yellowish green	12	14.12	0.36
	2	Green	65	76.47	
	3	Blue green	8	9.41	
Tendril color	1	Pale green	31	36.47	0.85
	2	Green	48	56.47	
	3	Purplish green	6	7.06	
Leaf size	1	Very small	6	7.06	0.65
	3	Small	25	29.41	
	5	Medium	37	43.53	
	7	Large	14	16.47	
	9	Very large	3	3.53	
Flower color	1	White	24	28.24	0.58
	2	Creamy white	59	69.41	
	3	Cream	2	2.35	
Immature pod color	1	Light green	14	16.47	0.76
	3	Slight light green	9	10.59	
	5	Green	48	56.47	
	7	Slight dark green	12	14.12	
	9	Dark green	2	2.35	
Twining tendril	3	Low	20	23.53	0.79
	5	Intermediate	51	60.00	
	7	High	14	16.47	
Growth pattern	1	Flat	22	25.88	0.49
	2	Erect	63	74.12	
Pod texture	1	Rough	65	76.7	0.38
	2	Smooth	12	14.16	
	3	Tuberculate	8	9.44	
Pod curvature	1	Absent	12	14.12	0.89
	3	Weak	62	72.94	
	5	Medium	9	10.59	
	7	Strong	2	2.35	
	9	Very Strong	0	0.00	
Seed size	1	Small	17	20.00	0.68
	2	Medium	62	72.94	
	3	Large	6	7.06	
Seed color	1	Cream	25	29.41	0.68
	2	Light yellow	16	18.82	
	3	Whitish green	44	51.76	
	4	Green	0	0.00	
	9	Others	0	0.00	
Seed wrinkle	1	Absent	13	15.29	0.41
	9	Present	72	84.71

### Genotypic variations in agro-morphological traits

The combined analysis of variance over two years data revealed significant differences for studied traits among the tested field pea genotypes (Table 4). However, the analysis of variance for year showed non-significant differences among the tested genotypes (Table 4). For this reason, the data of years 2022 and 2023 were pooled and the pooled data were analyzed for diversity study and other multivariate analysis. The results of the wide range of parameters for mean performance indicated that significant variations were seen in all of the investigated features, particularly in yield, seed size, pod setting, and disease response. Table 5 displays the average values for each characteristic in the 85 genotypes of field peas that were studied. For genotypes BFP30 and BFP56, the DF was found to have a mean of 54.88 days and a range of 25–91 days, respectively (Tables 5 and 6; Supplementary Tables S1 & S3). The DM values, which varied from 84 to 112 days (BFP84 to BFP57 and BFP61), had grand averages of 103.14 (Tables 5 and 6). From the vegetative stage until maturity, the plant's height ranges from 11.94 to 219.62 cm, with a mean height of 113.68 cm (Tables 5 and 6; Supplementary Tables S1 & S3). The PPP substantially distinguished across genotypes, with BFP65 (6.3) and BFP74 (47.78), respectively, having the highest and the lowest values, while the mean was 21.13 (Tables 5 and 6; Supplementary Tables S1 & S3). The SPP and PM had grand means of 4.87 and 3.71, with a range of 5.32 to 6. BFP04 had the highest PL (6.14), followed by BFP55, BFP65, and BFP14 (Tables 5 and 6; Supplementary Tables S1 & S3). Between genotypes, significant variance in HSW was displayed. The lowest HSW was shown by the BFP50, while the highest HSW and mean were shown by the BFP44 (Tables 5 and 6; Supplementary Tables S1 & S3). Field pea genotypes' YPP varied from 0.75 to 26.16 g. BFP78 provided the greatest YPP, followed by BFP77, BFP74, BFP72, BFP45, and BFP63. BFP77, BFP74, BFP72, and BFP63 were highly resistant to resistant and high yielding. The genotypes BFP78, BFP79, and BFP48, on the other hand, were highly productive and only moderately resistant. BFP44, however, demonstrated high production potential and moderate susceptibility (Table 7 Supplementary Tables S2 & S4).

**Table 4:** Combined analysis of variance (ANOVA) for yield and different agro-morphological traits for eighty-five filed peas genotypes studied over two years.

	Traits	Source of variation				
		Year (df = 1)	Replication within year (df = 4)	Genotype (df = 84)	Genotype × year (df = 84)	Pooled error (df = 338)
Mean Squares	DF	395.2	12.14	1103.9***	0.3	0.4
	DM	31.6	38.39	182.51***	0.01	1.15
	PH	909	3.77	8644***	2.0	12.0
	PPP	35.5	20.2	442.2***	0.1	0.9
	SPP	23.31	5.37	5.34***	0.02	0.015
	PL	1.04	35.68	1.55***	2.13	0.02
	HSW	1.15	9.75	437.4***	0.02	0.3
	PM	1.05	2.70	324.67***	0.04	0.9
	YPP	1.03	1.43	835.5***	0.01	1.7

DF = days to 80% flowering, DM = days to maturity, PH = plant height, PPP = pods per plant, SPP = seeds per pod, PL = pod length (cm), HSW = hundred seed weight, PM = powdery mildew and YPP = yield per plant, ***Significant at *p* < 0.01 level.

**Table 5:** The average performance of the genotypes for the characters (Pooled values of 2022 and 2023).

Code	DF	DM	PH	PPP	SPP	PL	HSW	YPP	PM
BFP01	56	106	129.04	18.91	5.96	5.50	9.84	9.02	5
BFP02	55	103	140.87	29.08	4.90	4.66	10.61	12.68	5
BFP03	41	99	113.54	9.76	5.54	5.34	13.59	5.59	4
BFP04	55	95	112.11	12.41	7.45	6.14	10.11	7.41	3
BFP05	45	98	98.03	14.03	5.96	5.07	8.50	5.37	7
BFP06	48	98	107.42	20.54	4.26	4.55	11.06	7.72	3
BFP07	33	87	25.00	16.68	5.11	4.97	11.37	7.73	6
BFP08	50	97	97.01	16.27	4.90	4.66	7.00	3.97	5
BFP09	53	96	92.79	18.30	5.98	5.05	5.84	4.69	6
BFP10	54	95	103.13	26.03	6.17	5.14	6.73	8.75	4
BFP11	54	107	139.65	13.83	4.69	4.86	18.89	10.05	4
BFP12	53	105	91.10	19.32	5.75	5.24	5.92	4.88	4
BFP13	54	104	118.84	23.79	5.90	5.09	7.91	9.12	3
BFP14	55	105	98.64	22.98	6.60	5.62	5.75	6.84	5
BFP15	44	95	123.53	23.39	4.90	4.91	10.79	10.16	4
BFP16	56	102	120.68	30.91	5.02	4.86	10.63	13.57	5
BFP17	61	103	138.43	18.50	4.26	5.21	18.03	11.85	3
BFP18	43	105	99.66	27.86	3.41	4.07	12.38	9.61	3
BFP19	54	106	101.30	16.06	4.79	4.29	5.88	3.10	4
BFP20	41	97	124.35	14.24	4.85	4.38	12.02	6.53	3
BFP21	40	103	122.92	32.33	4.69	4.96	13.86	18.04	2
BFP22	60	104	135.77	21.76	5.32	5.03	9.92	9.37	4
BFP23	47	98	128.63	22.98	3.83	3.93	11.36	8.02	3
BFP24	54	95	104.56	22.37	5.32	4.83	6.14	5.55	4
BFP25	41	103	98.44	20.95	4.04	4.19	12.81	8.79	4
BFP26	54	102	99.25	21.15	5.15	4.41	6.03	4.83	4
BFP27	57	104	171.68	22.78	5.11	5.14	9.91	9.40	4
BFP28	41	92	115.78	17.89	4.69	4.51	13.17	8.96	3
BFP29	48	102	123.33	28.26	5.12	5.16	11.39	13.90	2
BFP30	25	100	27.65	6.51	3.62	4.23	20.36	3.26	2
BFP31	45	106	102.11	18.10	3.41	4.07	18.75	9.43	2
BFP32	55	100	113.95	26.23	5.75	5.22	6.76	8.18	3
BFP33	63	102	122.92	16.47	4.26	4.45	10.27	5.46	2
BFP34	56	100	112.14	16.68	4.47	4.55	10.69	6.16	2
BFP35	54	100	109.25	13.22	5.97	5.40	12.39	7.79	2
BFP36	46	102	118.43	17.08	4.47	4.40	9.01	5.17	5
BFP37	49	95	105.58	20.74	5.32	4.67	11.39	10.36	3
BFP38	50	95	90.89	18.91	4.69	4.44	6.32	3.99	3
BFP39	48	106	99.05	24.81	5.54	4.97	11.79	13.67	3
BFP40	56	107	103.54	25.42	5.11	4.77	7.22	7.44	3
BFP41	53	107	185.14	20.95	5.58	5.03	9.40	8.83	3
BFP42	51	106	101.09	24.40	4.26	4.35	6.20	4.76	3
BFP43	43	100	97.62	20.13	5.32	5.58	18.07	16.57	3
BFP44	57	108	145.16	31.11	4.90	5.02	76.34	15.07	5
BFP45	56	106	173.92	32.13	4.04	5.15	20.64	23.38	4
BFP46	53	105	116.19	24.60	6.39	5.51	5.48	6.75	4
BFP47	68	106	145.56	25.82	4.47	5.01	13.79	13.42	6
BFP48	57	110	117.62	17.89	4.90	5.25	22.94	17.24	4
BFP49	63	105	131.08	16.88	5.75	5.44	15.37	12.49	6
BFP50	84	109	86.60	9.96	5.53	4.88	3.72	0.75	4
BFP51	64	107	132.92	22.78	4.47	4.96	11.36	9.44	6
BFP52	78	110	118.74	19.93	3.41	3.87	22.80	13.02	2
BFP53	86	110	78.85	13.83	3.83	4.74	8.76	3.12	4
BFP54	59	107	104.36	13.22	4.26	4.85	16.39	7.30	4
BFP55	68	108	82.32	14.44	6.17	5.91	8.42	5.73	3
BFP56	91	110	89.67	12.81	4.04	4.09	7.23	2.30	6
BFP57	86	112	85.18	10.17	4.69	5.11	10.15	3.29	3
BFP58	57	108	53.97	13.42	5.75	5.53	17.09	10.92	5
BFP59	76	107	38.05	11.59	6.12	5.45	9.65	5.19	3
BFP60	40	106	75.39	9.96	5.54	5.48	13.69	5.78	6
BFP61	80	112	115.17	15.25	2.13	2.98	18.49	4.36	4
BFP62	67	107	131.29	25.62	5.09	5.53	15.72	17.67	3
BFP63	46	108	11.94	32.74	4.69	4.86	15.34	20.35	3
BFP64	63	106	78.85	13.42	3.41	4.28	22.90	8.44	5
BFP65	67	108	94.36	6.30	6.39	5.86	13.60	3.88	5
BFP66	53	105	107.82	16.88	4.04	4.34	17.45	9.76	6
BFP67	30	88	87.63	10.37	5.32	5.03	17.51	7.71	3
BFP68	48	104	87.83	16.88	5.96	5.29	6.75	5.08	3
BFP69	84	98	87.63	9.56	2.77	4.31	14.11	2.29	3
BFP70	83	100	86.00	15.66	4.04	4.64	16.89	8.65	4
BFP71	86	101	73.55	10.37	3.83	4.82	14.67	4.21	3
BFP72	71	109	143.73	26.23	3.83	5.24	27.38	24.01	1
BFP73	63	108	101.09	20.13	6.60	5.46	4.93	4.86	2
BFP74	53	107	180.66	47.78	6.06	5.30	9.58	24.67	3
BFP75	56	107	192.08	37.62	3.62	4.63	13.78	16.01	4
BFP76	49	107	219.62	44.94	4.47	4.96	8.81	15.05	4
BFP77	49	106	182.62	43.11	4.69	5.11	14.25	25.15	3
BFP78	48	105	181.47	41.48	4.90	5.05	14.71	26.16	4
BFP79	54	105	197.79	31.52	4.26	5.05	15.84	18.29	4
BFP80	45	106	192.20	25.62	4.47	4.95	16.81	16.46	3
BFP81	66	106	154.75	33.35	4.90	4.51	5.82	7.55	2
BFP82	67	107	83.55	20.74	3.62	4.58	18.23	11.37	3
BFP83	56	103	119.86	28.26	4.47	4.58	5.80	5.57	4
BFP84	28	84	66.41	16.27	4.04	4.95	16.24	8.64	2
BFP85	51	104	144.75	32.13	4.85	4.85	10.63	14.15	4
LSD (0.05)	0.72	1.22	3.93	1.08	0.14	0.15	0.62	1.48	0.12
CV (%)	1.13	1.04	3.05	4.49	2.51	2.76	4.25	12.15	1.09
LS	***	***	***	***	***	***	***	***	***

DF = days to 80% flowering, DM = days to maturity, PH = plant height, PPP = pods per plant, SPP = seeds per pod, PL = pod length (cm), HSW = hundred seed weight, PM = powdery mildew and YPP = yield per plant.

**Table 6:** The descriptive statistics and analysis of eight quantitative morphological traits of field pea genotypes. (Pooled values of 2022 and 2023).

Variables	Min	Max	Range	Mean ± SE	CV	H'
DF	25	91	66	55.85 ± 1.46	1.13	1.00
DM	84	112	28	103.14 ± 0.6	1.04	1.91
PH	11.94	219.62	207.68	113.68 ± 4.12	3.05	3.62
PPP	6.3	47.78	41.48	21.13 ± 0.93	4.49	1.12
SPP	2.13	7.45	5.32	4.87 ± 0.1	2.51	0.80
PL	2.98	6.14	3.16	4.89 ± 0.06	2.76	1.93
PM	1	7	6	3.71 ± 0.13	1.09	0.45
HSW	3.72	76.34	72.62	12.9 ± 0.93	4.25	0.88
YPP	0.75	26.16	25.41	10.73 ± 1.28	12.15	1.23

H' = Shannon Weaver diversity index, DF = days to 80% flowering, DM = days to maturity, PH = plant height, PPP = pods per plant, SPP = seeds per pod, PL = pod length (cm), HSW = hundred seed weight, PM = powdery mildew and YPP = yield per plant.

**Table 7:** Response of different field pea genotypes at different growth stages screened against powdery mildew (*Erysiphe polygoni*) (Pooled values of 2022 and 2023).

Entry code	Disease				
	Severity at early stage (1–9)	Severity at flowering stage	Severity at pod setting	Average	Response
BFP01	4	5	8	5	MS
BFP02	3	5	7	5	MS
BFP03	4	5	5	4	MR
BFP04	3	4	3	3	R
BFP05	5	7	9	7	S
BFP06	3	3	4	3	R
BFP07	5	6	8	6	MS
BFP08	3	5	8	5	MS
BFP09	4	6	7	6	MS
BFP10	4	4	5	4	MR
BFP11	3	4	4	4	MR
BFP12	4	4	4	4	MR
BFP13	2	3	3	3	R
BFP14	4	5	6	5	MS
BFP15	2	5	5	4	MR
BFP16	4	6	6	5	MR
BFP17	3	3	3	3	R
BFP18	3	3	3	3	R
BFP19	3	5	5	4	MR
BFP20	3	3	3	3	R
BFP21	3	2	2	2	HR
BFP22	4	4	4	4	MR
BFP23	2	3	3	3	R
BFP24	3	5	5	4	MR
BFP25	3	5	6	4	MR
BFP26	3	4	5	4	MR
BFP27	3	4	5	4	MR
BFP28	2	3	3	3	R
BFP29	2	2	3	2	HR
BFP30	2	2	2	2	HR
BFP31	2	2	2	2	HR
BFP32	3	3	4	3	R
BFP33	2	2	3	2	HR
BFP34	3	2	3	2	HR
BFP35	2	2	3	2	HR
BFP36	4	5	6	5	MS
BFP37	3	4	4	3	R
BFP38	3	3	4	3	R
BFP39	3	3	3	3	R
BFP40	2	4	5	3	R
BFP41	3	3	4	3	R
BFP42	3	3	4	3	R
BFP43	3	4	3	3	R
BFP44	5	5	7	5	MS
BFP45	4	4	4	4	MR
BFP46	3	3	5	4	MR
BFP47	5	6	7	6	MS
BFP48	3	4	4	4	MR
BFP49	5	6	7	6	MS
BFP50	3	4	4	4	MR
BFP51	6	6	7	6	MS
BFP52	1	2	2	2	HR
BFP53	3	4	4	4	MR
BFP54	4	4	5	4	MR
BFP55	3	4	3	3	R
BFP56	5	6	7	6	MS
BFP57	2	3	4	3	R
BFP58	4	5	6	5	MS
BFP59	2	3	4	3	R
BFP60	6	6	8	6	MS
BFP61	3	4	3	4	MR
BFP62	3	4	3	3	R
BFP63	3	3	3	3	R
BFP64	3	5	6	5	MS
BFP65	3	5	6	5	MS
BFP66	4	6	6	6	MS
BFP67	2	3	3	3	R
BFP68	3	3	3	3	R
BFP69	2	4	4	3	R
BFP70	3	4	4	4	MR
BFP71	2	3	3	3	R
BFP72	1	2	2	2	HR
BFP73	2	2	3	2	HR
BFP74	2	2	4	3	R
BFP75	3	4	4	4	MR
BFP76	3	4	5	4	MR
BFP77	3	3	3	3	R
BFP78	3	4	5	4	MR
BFP79	3	4	4	4	MR
BFP80	3	4	3	3	R
BFP81	2	3	3	2	HR
BFP82	3	3	4	3	R
BFP83	3	4	5	4	MR
BFP84	1	2	2	2	HR
BFP85	3	4	4	4	MR

### Shannon Weaver Diversity analysis combined with descriptive statistics

The diversity of the accessions on the quantitative characteristics shown in Table 6 was assessed using the descriptive statistics (average, range, and standard deviation) and Hʹ. All of the genotypes' coefficients of variation fell into two categories: medium (between 10 and 20%), or low (below 10%). The ninequantitative features' coefficients of variance varied from 1.04 to 12.15%. The YPP population had the highest CV (12.15%), followed by PPP (4.49), HSW (4.25), and PH (3.05). The field pea genotype's high intra-varietal variability was demonstrated by the larger coefficients of variation for PH, PL, and DM. SPP (2.13–7.45) had the lowest diversity index (0.80) across morphological traits while PH (11.94–219.62 cm) displayed the greatest diversity value (3.62). A high diversity index (H') was also seen in the yield-related characteristics DF (1.00), DM (1.91), PPP (1.12), PL (1.93), HSW (0.88), and YPP (1.23).

### Response of genotypes to the illness of powdery mildew

**Figure 3:**
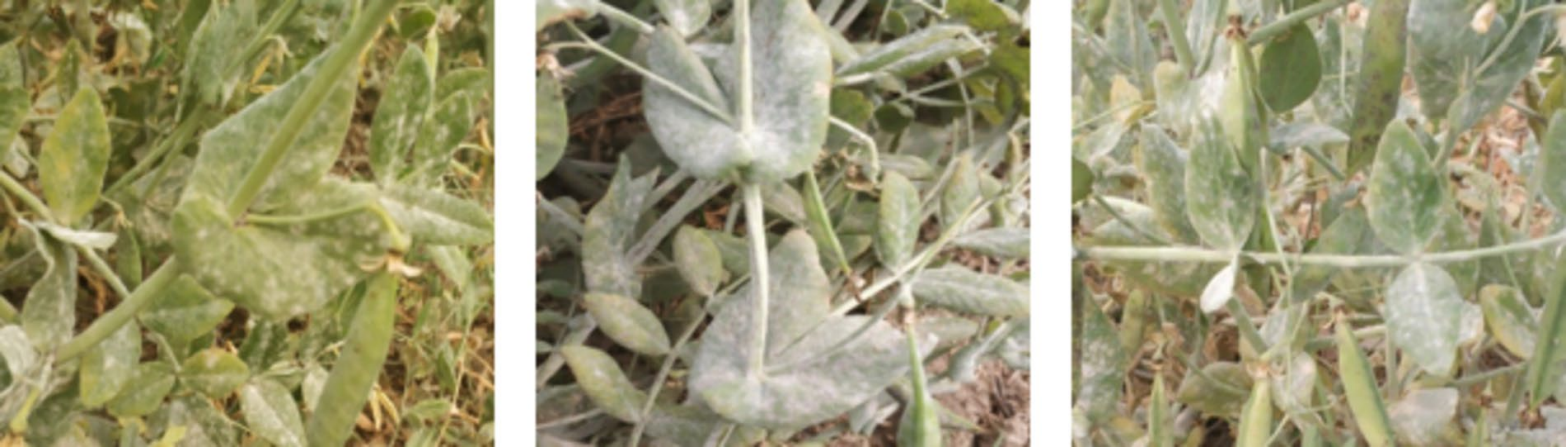
Infection level and severity of powdery mildew disease in field pea genotypes.

The genotypes of field peas were tested in the field at three development stages for natural infection with the powdery mildew disease caused by *Erysiphe polygoni*. From the early stages to flowering and pod setting, the disease's severity increased (Table 7, Supplementary Tables S2 & S4, and Fig. 3). The responses of all genotypes examined to the illness caused by powdery mildew varied greatly. As a result, among the genotype sources, 14.11 and 34.11% of the genotypes, respectively, were found to be highly resistant to *E. polygony* infection. Hence forward, it was found that out of the total 85 field pea genotypes, twelve genotypes (BFP21, BFP29, BFP30, BFP31, BFP33, BFP34, BFP35, BFP52, BFP72, BFP73, BFP81 and BFP84) i.e. 14.11% were highly resistant (Disease Severity Scale 2) (Table 8**)**, twenty-nine (BFP04, BFP06, BFP13, BFP17, BFP18, BFP20, BFP23, BFP28, BFP32, BFP37, BFP38, BFP39, BFP40, BFP41, BFP42, BFP43, BFP55, BFP57, BFP59, BFP62, BFP63, BFP67, BFP68, BFP69, BFP71, BFP74, BFP77, BFP80 and BFP82) i.e. 34.11% were resistant (Disease Severity Scale 3), twenty-five (BFP03, BFP10, BFP11, BFP12, BFP15, BFP19, BFP22, BFP24, BFP25, BFP26, BFP27, BFP45, BFP46, BFP48, BFP50, BFP53, BFP54, BFP61, BFP70, BFP75, BFP76, BFP78, BFP79, BFP83 and BFP85) i.e. 29.41% were moderately resistance (Disease Severity Scale 4), eighteen (BFP01, BFP02, BFP08, BFP14, BFP16, BFP36, BFP44, BFP58, BFP64, BFP65, BFP07, BFP09, BFP47, BFP49, BFP51, BFP56, BFP60 and BFP66) i.e. 21.76% were moderately susceptible (Disease Severity Scale 5 & 6) and three (BFP05) i.e. 1.17% were susceptible (Disease Severity Scale 7) (Table 8 and Fig. 3). The genotypes BFP21, BFP30, BFP31, BFP33, BFP34, BFP52, BFP72 were highly resistance along with check variety BFP84 (BARI Motor-2).

**Table 8:** Disease response, frequency, and percentage of the field pea genotypes screened against *Erysiphe polygoni* (Pooled values of 2022 and 2023).

Response	Disease severity scale (1- 9)	Genotypes	Frequency	Percentage
Immune	1	No	-	
Highly resistant	2	BFP21, BFP29, BFP30, BFP31, BFP33, BFP34, BFP35, BFP52, BFP72, BFP73, BFP81 and BFP84	12	14.11
Resistant	3	BFP04, BFP06, BFP13, BFP17, BFP18, BFP20, BFP23, BFP28, BFP32, BFP37, BFP38, BFP39, BFP40, BFP41, BFP42, BFP43, BFP55, BFP57, BFP59, BFP62, BFP63, BFP67, BFP68, BFP69, BFP71, BFP74, BFP77, BFP80 and BFP82	29	34.11
Moderately resistant	4	BFP03, BFP10, BFP11, BFP12, BFP15, BFP19, BFP22, BFP24, BFP25, BFP26, BFP27, BFP45, BFP46, BFP48, BFP50, BFP53, BFP54, BFP61, BFP70, BFP75, BFP76, BFP78, BFP79, BFP83 and BFP85	25	29.41
Moderately susceptible	5 & 6	BFP01, BFP02, BFP08, BFP14, BFP16, BFP36, BFP44, BFP58, BFP64, BFP65, BFP07, BFP09, BFP47, BFP49, BFP51, BFP56, BFP60 and BFP66	18	21.76
Susceptible	7	BFP05	1	1.17
Highly susceptible	8	No	–	
Highly susceptible	9	No	–	

### Principal component analysis (PCA)

**Figure 4:**
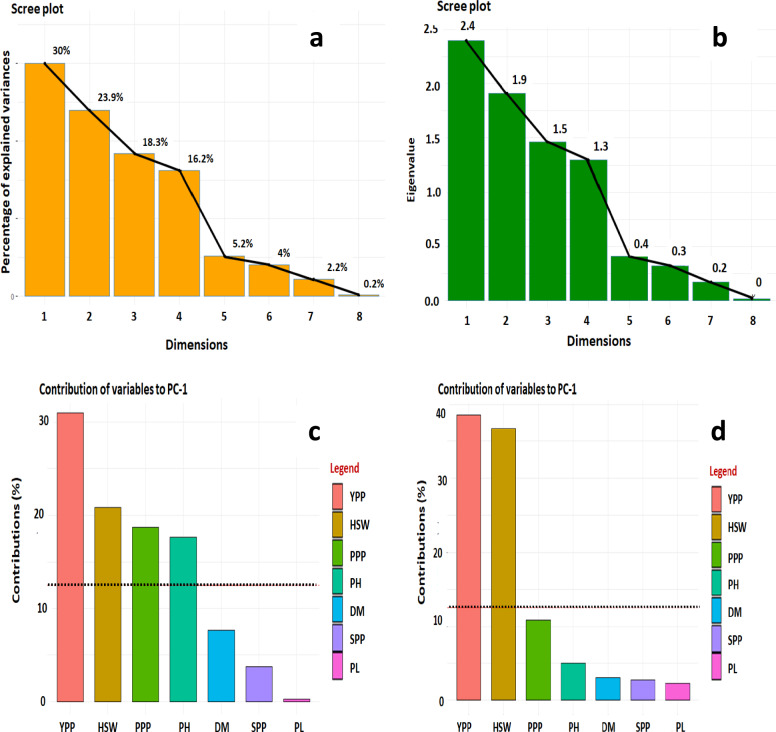
(**a**) Proportion of variance (%) of top 8 principal components (PCs), (**b**) Eigenvalues of top 8 PCs, (**c**) Contribution of variables to PC1 (%), and (**d**) Contribution of variables to PC2 (%) derived from principal component analysis (PCA). Red dashed lines across bar plots are the reference lines and the variable bars above the reference lines are considered—important in contributing to the respected PCs.

The results of PCA showed that only the first four principal components (PCs) had eigenvalues greater than 1.00 and that the highest variability across field pea genotypes for yield component attributes was around 88.4% (Fig. 4a,b). In field pea improvement programs, the traits corresponding to these five PCs may be given the appropriate weight. Nine characteristics were used for the principal component analysis (Fig. 4b). 72.2% of the variance was explained by the first three principal components (PC), which had values of 30, 23.9, and 18.3% for PC1, PC2, and PC3, respectively (Fig. 4a). Following the PC1 in terms of variety were the PC2, PC3, PC4, and PC5. The earlier research by Hanci and Cebeci^83^ provided further support for the current investigation. The decision of how many variables to keep is aided by eigenvalues. According to Sharma^54^, the number of variables is often equal to the sum of the eigenvalues. The first principal component (PC1) exhibited a variance of 30%, with the key positive contributions being YPP, HSW, PH, and PPP, while the significant negative contributors were PL, DM, and SPP (Fig. 4c). The PC2 was mostly linked to yield parameters including DF, DM, and PPP, and accounted for 23.9% of the overall variance (Fig. 4d).

**Figure 5:**
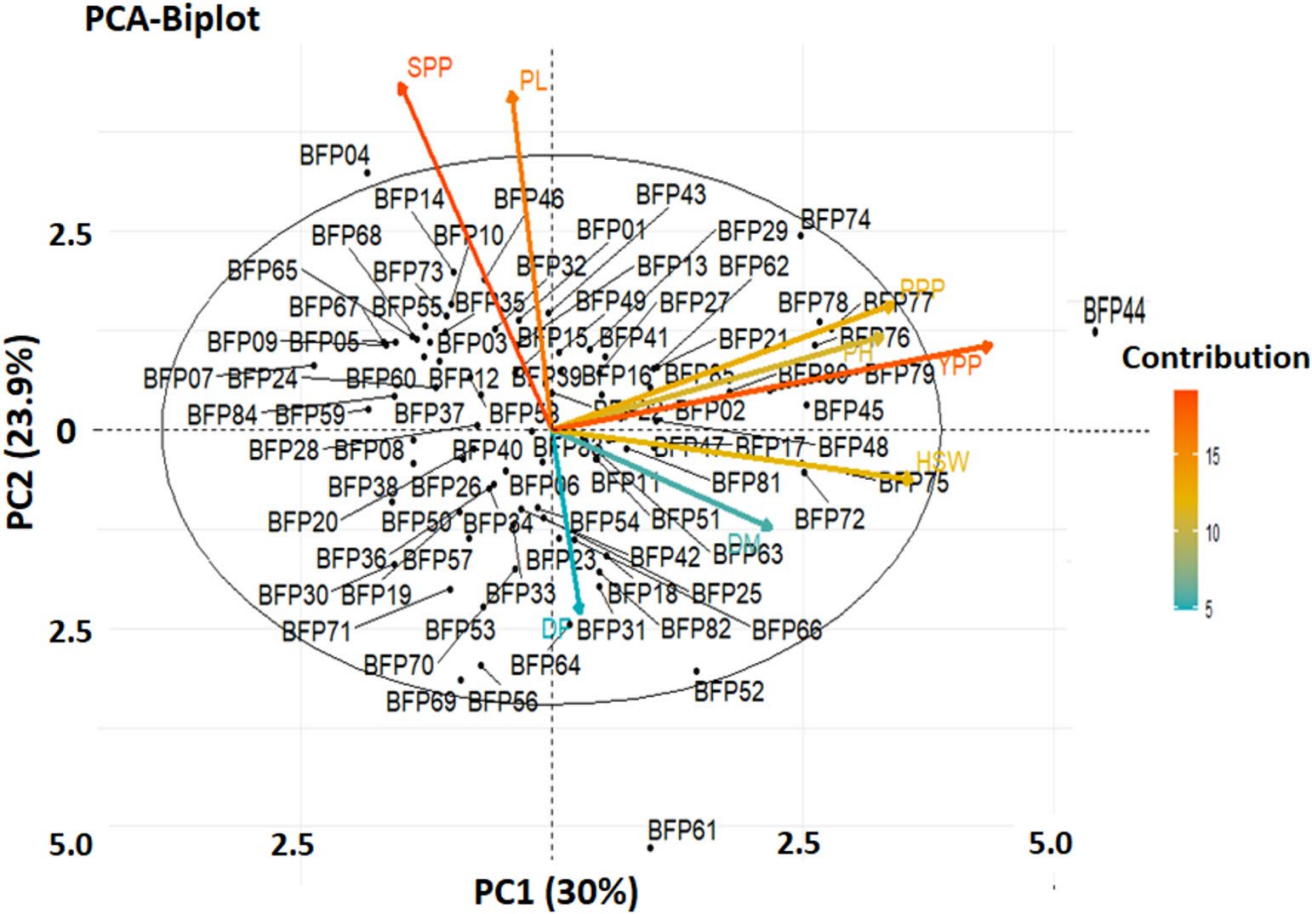
shows a biplot of the PCA method that demonstrates the association between measured traits and field pea genotypes. Five groups of the 85 field pea genotypes are represented by different colors of the individuals (genotypes). The magnitude of the overall contribution of the variables to PC1 and PC2 is shown by the length and color intensity of the arrows. PC1 on the x-axis provided 30% of the overall variability, whereas PC2 on the y-axis contributed 23.9% of the total variability.

Figure 5 depicts the magnitude and direction of the quantitative features' contribution to the various main components. The PCA-biplot was created using the first two PCs, which together accounted for 53.9% of the total variability (Fig. 5). The key yield and yield-attributing traits, however, grouped in trait clusters 2 and 3, such as HSW, PL, and YPP, were mostly connected with and positively contributed to PC2 and PC3 (Figs. 4c,d, 5). Based on the features that contribute to yield, the genotypes in clusters 4 and 5 dominated substantially and were favorably emphasized in both PC1 and PC2 (Fig. 5); in contrast, the genotypes in cluster 3 were strongly reflected by the traits DF, DM, and PL (Fig. 4).

### Analysis of cluster distances

**Figure 6:**
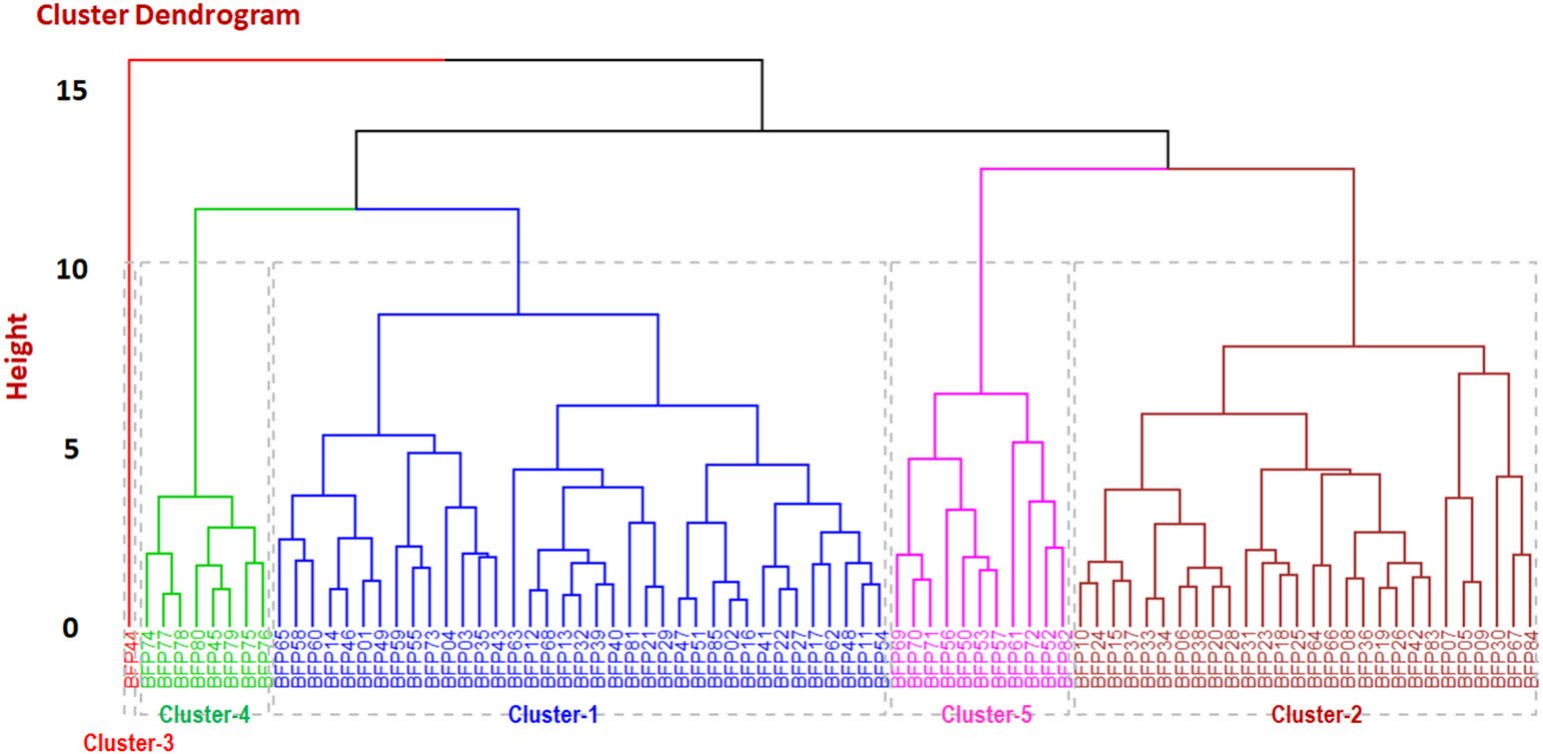
Agglomerative hierarchical clustering (AHC) dendrogram analysis using Euclidean distance into different clusters by the Ward method for quantitative morphological traits of 85 field pea genotype.

Based on D^2^ values, cluster analysis was used to divide the 85 genotypes into 5 distinctive groups. The number of genotypes in each cluster ranged from 1 to 37. Cluster 1 included the greatest number of genotypes (37) possible. Cluster 3 included the fewest number of genotypes only one. The genotypes in clusters 2, 4, and 5 were 28, 8, and 11, respectively (Table 9 and Fig. 6). Prasad et al.^1^ and Kumar et al.^84^ noticed that great variety was present in the material under evaluation, as evidenced by the discrimination of genotyping lines into so many distinct clusters. Table 9 and Fig. 6 provide estimates of intra- and inter-cluster distances for five clusters. Cluster 5 had the highest intra-cluster value (3.40), followed by Cluster 2 (3.01), Cluster 1 (2.92), and Cluster 4 (2.06), indicating that these clusters' genotypes exhibit substantial genetic diversity (Table 9). Cluster 2 and Cluster 3 had the greatest inter-cluster distance (11.89), followed by Cluster 1 and Cluster 3 (11.43) and Cluster 3 and Cluster 5 (5.35), indicating the greatest genotypic diversity in these clusters. Therefore, it is suggested that improved segregants for high seed production and yield-contributing characteristics owing to non-allelic interactions are predicted if different genotypes from these groups are employed in breeding programs along with other desired features. Cluster 3 and cluster 4 exhibited the least genetic diversity among their clusters, and they had the same genetic architecture, as shown by the smallest inter-cluster difference between them (2.06), which was followed by cluster 4 and cluster 5 (3.40) (Table 9). In order to disrupt the unfavorable relationship between yield and its associated qualities, such genotypes may also be employed in breeding programs to create bi-parental crosses between the most diversified and close-proximity groups.

**Table 9:** Average intra- and inter-cluster distances for five clusters in field pea genotype.

Cluster numbers	Intra-cluster distance	Inter-cluster distance				
		1	2	3	4	5
1	2.92					
2	3.01	3.61				
3	0	11.43	11.89			
4	2.06	3.95	4.61	2.06		
5	3.40	4.39	4.44	5.35	3.40

### Genotypes are grouped using a heatmap-oriented clustering pattern

**Figure 7:**
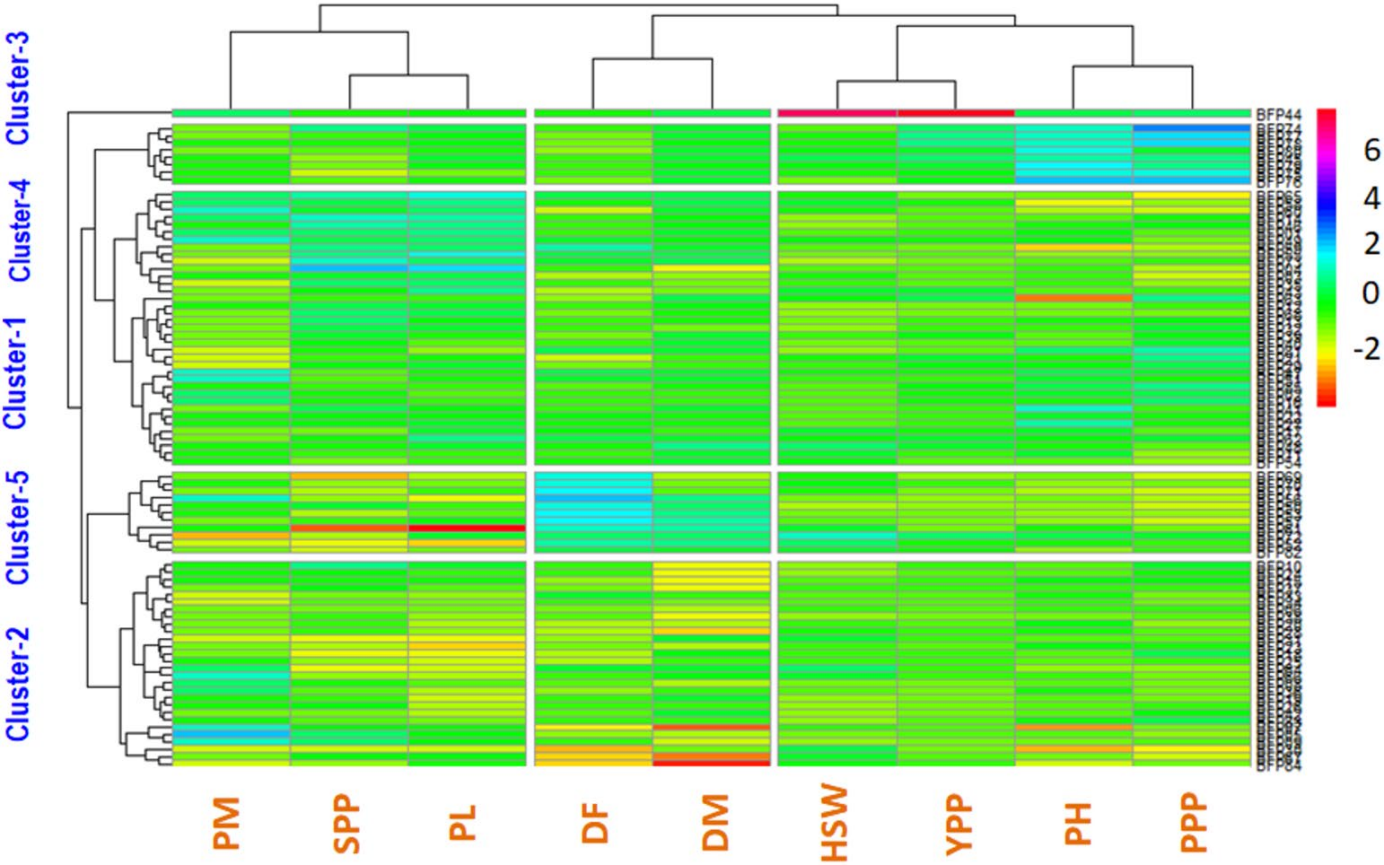
The grouping pattern of 85 field pea genotypes with 8 quantitative features is shown on the heatmap. Each row denotes a genotype, whereas each column denotes a character. Based on the link between the genotype and the characteristics, the different colors and intensities (- 2 to 6) were modified. The colors red and green stand for lower values, blue for higher values, and green for mid-values.

A heatmap is a two-dimensional data visualization technique that uses color to show the size of a phenomenon. By examining color variation by intensity, the reader may observe how the phenomenon is categorized or varies through time. On a backdrop of mostly minor features, it depicts the relative distribution of strongly expressed qualities (Figs. 6 and 7). The heatmap analysis produced two dendrograms as a consequence, one in the vertical direction representing the germplasm accessions and the other in the horizontal direction reflecting the attributes that caused the diffusion. A heatmap is a two-dimensional data visualization technique that uses color to show the size of a phenomenon. Eighty-five field pea genotypes were used in the current research, and using heatmap-oriented cluster analysis, the genotypes were divided into five groups based on the average values of all the analyzed variables. The distribution of genotypes in the clusters showed that cluster 1 had the highest number of genotypes (37) and cluster 3 had the lowest number of genotypes (1) (Table 10 and Figs. 6 and 7). Three other groups might be seen on other dendrograms. DF and DM are two characters connected to Group 1. Three characters (YPP, HSW, and PL) are related to Group 3. Three characters (SPP, PH, and PPP) are allies of Group 3 (Fig. 7).

**Table 10:** Cluster mean values for different characters in field pea genotype.

Cluster number	Number of genotypes	DF	DM	PH	PPP	SPP	PL	HSW	YPP	PM
1	37	55.86	104.76	113.21	20.90	5.46	5.23	11.59	10.05	3.76
2	28	47.04	98.43	98.58	18.67	4.61	4.55	11.21	6.87	3.75
3	1	57.00	108.00	145.16	31.11	4.90	5.02	76.34	15.07	5.00
4	8	51.25	106.13	190.05	38.03	4.56	5.03	14.30	20.65	3.63
5	11	81.45	107.09	95.33	14.96	3.79	4.48	14.77	7.03	3.36

DF = days to 80% flowering, DM = days to maturity, PH = plant height, PPP = pods per plant, SPP = seeds per pod, PL = pod length (cm), HSW = hundred seed weight, PM = powdery mildew and YPP = yield per plant.

### Analysis of clustered means

To determine the acceptable genotypic diversity present across all study groups, a dendrogram of 85 field pea genotypes was created using the Ward clustering technique^85^. The significant degree of variation in cluster means for several traits (Table 10) further supported the variety. Eight yield and yield contributing characteristics, along with PM cluster means, were evaluated (Table 10). The average comparison of the various characters revealed significant variations among the clusters for each character. Cluster 5 had the highest mean for DF (81.45), followed by Cluster 3 (57), while Cluster 2 had the lowest mean for a DF (47.04). The mean values for Cluster 3 were the greatest for DM, HSW, and PM. In cluster 4, the greatest means for PH (190.05), PPP (38.03), and YPP (20.65) were found. Twenty-eight genotypes constituted the Cluster 2, which had the second highest number of genotypes. This cluster had smaller seeds than the others, was more moderately vulnerable to powdery mildew, and had a lower YPP than the others. In cluster 2, none of the characters had the highest mean value. These results showed that certain clusters performed better for various character types.

### Analysis of the trait associations

**Figure 8:**
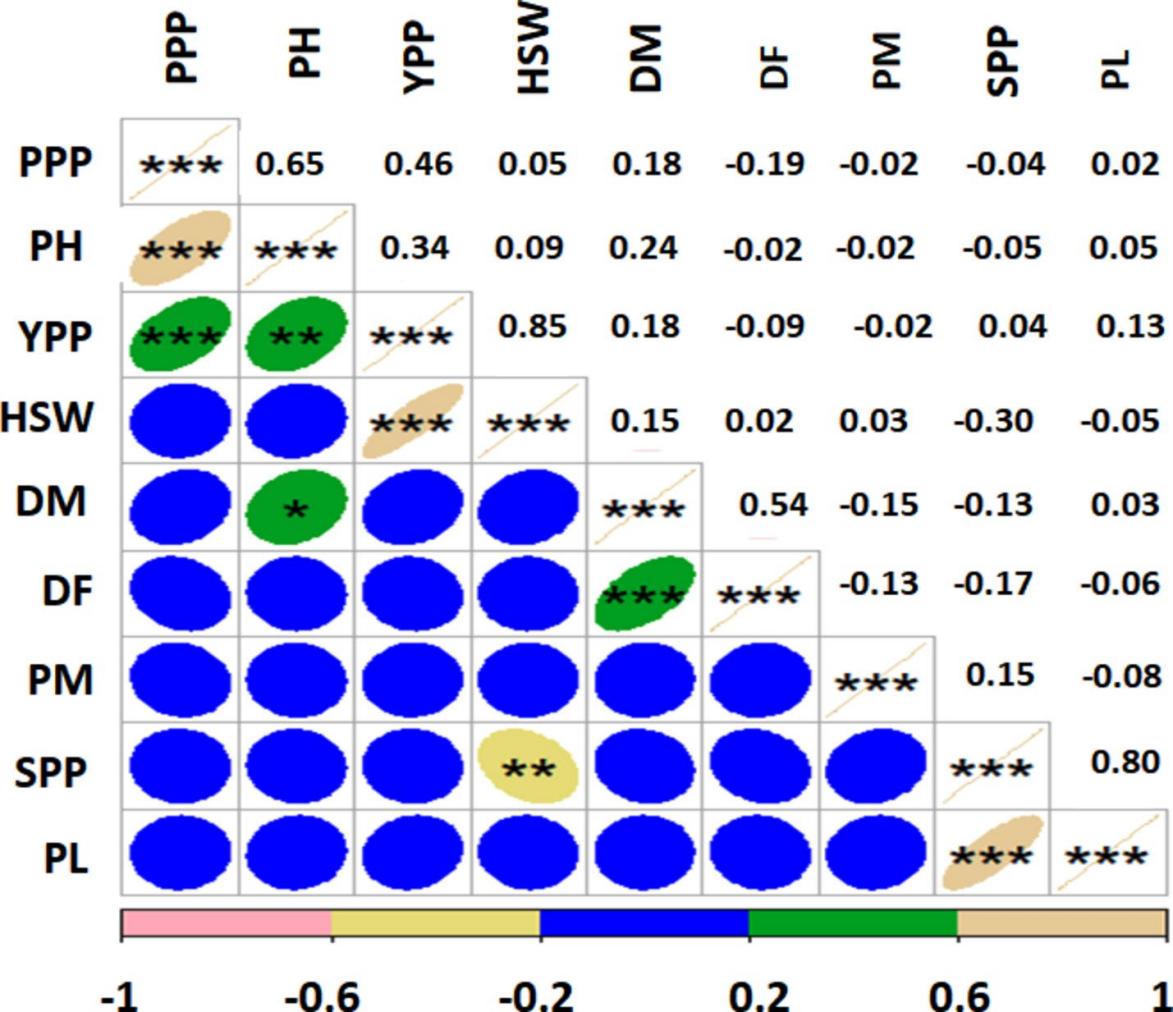
Correlations among the traits scored; Pearson’s rank correlation matrix and performance analytic chart of the variables showing the relationship among the variables scored on field pea accessions. PH = plant height, DF = days to 80% flowering, DM = days to maturity, PL = pod length (cm), PPP = pods per plant, SPP = seeds per pod, PM = Powdery mildew, HSW = hundred seed weight, and YPP = yield per plant. *, ** and *** = Significant at *p* < 0.05, *p* < 0.01 and *p* < 0.001, respectively.

There were very strong relationships among the traits that were assessed (Fig. 8). The coefficient of correlation is the measurement of the linear relationship between two variables. The correlation of nine parameters under field conditions is presented in Fig. 8. PPP had positive significantly correlation with PH (r = 0.65, *p* < 0.001), YPP (r = 0.46, *p* < 0.001), whereas it had negative nonsignificant correlation with DF, PM and SPP under field condition. PH demonstrated positive significant correlation with YPP (r = 0.34, *p* < 0.01), and DM (r = 0.24, *p* < 0.05) while expressed negligible negative correlation with DF, PM and SPP. YPP expressed positive highly significant correlation with HSW (r = 0.85, *p* < 0.001), while it showed negligible negative correlation with DF and PM. DF showed highly significant correlation with DM (r = 0.54, *p* < 0.001). Powdery mildew (PM) disease severity had nonsignificant positive correlation with HSW (r = 0.03) and SPP (0.15). whereas rest of the traits had a nonsignificant negative correlation with powdery mildew disease severity (Fig. 8). PL represented highly positive correlation with SPP (r = 0.80, *p* < 0.001).

## Discussion

An established crop breeding technique for managing and successfully using plant genetic resources is genotype evaluation and screening for desired traits^86^. The degree of genetic diversity in agro-morphological variables associated with yield determines the breeding strategy. Research on multivariate analysis and genetic diversity is essential for an effective genotype assessment. Plant genotypes exhibit a great degree of morphological variation. A great degree of diversity is used to create better cultivars of important crops. Crops that are less popular or aren’t used as much need to benefit from this development as well since they have a high degree of variability both inside and between their accessions (intra-variation) and within (inter-variation). All studied field pea genotypes showed a variation across several traits which define a wide array of variability among the traits. A similar kind of variability for traits in rice was recorded by several researchers^87,88^. Breeders may choose better lines for future development by using morphological characterization in diverse areas^89–91^. Analyzing morphological traits is a common method for determining genetic diversity for many crop species, including field peas. It is successfully used on a variety of crops, including *Pisum sativum*^73^, mungbean^48,92^, black gram^93^, amaranth^94–98^, Maize^99^, and field pea^17,22,23^.

The findings of this research support highly the Shannon–Weaver diversity indices across field pea populations for the qualitative attributes of tendril color, tendril twinning, immature pod color, flower color, and seed size. Shubha et al.^100^ and Rosero-Lombana et al.^101^ both found similar findings for field pea. Table 3 displays the results of the Shannon–Weaver diversity study for qualitative traits. Indicators of variety varied from 0.36 for leaf color to 0.89 for pod curvature. Seven qualitative traits in this research have a high variety index (H = 0.60). The results of this research showed that there was little variation in leaf color and pod curvature across field pea genotypes, in contrast to studies^73,102^. For the majority of the characteristics listed in Tables 4 and 5, a broad range of differences were observed. The findings are validated by other research^17,73,103^ and show that features like PH, HSW, DF, PPP, DM and YPP, showed a very wide variation in mean performance. There was a lot of phenotypic diversity in seed yield and related traits. The great degree of diversity in yield and its associated traits was highlighted by the mean performance in this research (Table 3), suggesting that future breeding programs will have more opportunities to make use of these traits^104^. In keeping with the findings of the current study, several researchers including those of our own earlier study^16,17^ showed significant heterogeneity in field pea yield and its associated characteristics. Significant differences for studied traits among the tested field pea genotypes might be due to the difference in genetic composition of the tested genotypes. This indicates that the tested genotypes have different potential for field pea crop production for studied characters which corroborated with the results of Mogiso^105^ and Gurmu^106^ in field pea. Significant variations across genotypes were also reported in literatures^107–112^ which supported the current findings. Crops that are affected by the powdery mildew disease have large yield losses^74,113,114^. Field pea breeding for resistance to powdery mildew needs effective disease screening techniques. When field peas are produced for seed, powdery mildew losses are greater because the disease becomes more severe as the crop matures^114^. No matter where they came from, natural sources of resistance with varying disease responses to powdery mildew in field peas have been found in the germplasm^32^. Crops suffering from powdery mildew disease experience significant losses of yield^114^. It is necessary to use the right disease-screening techniques while breeding field peas to resist powdery mildew. According to Teshome^75^, the powdery mildew disease significantly reduces the yield potential of field pea germplasm grown throughout the world by generating an 86% loss. Therefore, the greatest alternative for crop breeding is genetically based resistance to harmful diseases^32,75,115^. In order to choose powdery mildew-resistant lines for documentation and identification of top lines for widespread cultivation, the chosen pea lines were also assessed for the powdery mildew disease. Researchers have used a variety of approaches to screen for powdery mildew^29^,^75,115^, but artificial inoculation has been found to be the most reliable and effective^29,105,115,116^. In this study, the same eighty-five germplasms were tested in a field setting for resistance to powdery mildew. Out of the 85 germplasms listed in Tables 6 and 7, none were found to be extremely resistant (immune). However, 29 germplasms were observed to be resistant and 12 to be highly resistant. Eighteen genotypes demonstrated moderately susceptible, while the remaining twenty-five germplasms exhibited moderate resistance. The BFP05 genotype showed susceptible to PM disease. In order to better understand the level of genetic diversity and agronomic performance of resistant germplasm accessions, more information should be gathered^29,117,118^. The germplasm accessions, which have high levels of resistance and agronomic superiority, may minimize the time necessary to eradicate the undesired genes via repeated backcrossing by plant breeders. Three basic selection strategies including tandem selection, independent culling levels and index selection can be utilized for improvement of plants species in breeding programs. Tandem selection attempts to improve a breeding germplasm for several traits by selecting one trait at one time for several generations, then another trait is focused on for next breeding cycle^119^. Identification of diversity serves two roles: it helps to organize the identity and integrity of an ex-situ collection and it also provides a structure accessing this diversity for breeding efforts^120^.

PCA is a useful method for locating significant characteristics that have a bigger influence on the total variables, and each vector's coefficient indicates the percentage contribution of each original variable that each principal component is linked to Sanni et al*.*^121^. According to studies, the first three main components are often the most significant in showing the patterns of variation among the various genotypes and the traits linked to genotype differentiation. Raji^122^ asserted that traits with coefficients greater than 0.3 (regardless of whether they are positive or negative) are important, while traits with coefficients less than 0.3 are thought to have the least impact on the overall variation seen. This methodology was used in the current study^123^.

Based on the dendrogram produced by cluster analysis and PCA, the 85 genotypes of field pea were divided into five groups (Tables 9 and 10 and Fig. 6). Sharma^103^ determined a similar clustering pattern using PCA and hierarchical cluster analysis for 22 field pea genotypes. According to Euclidean distance, clusters mostly emerge following the origin of genotypes or geographical locations. Most genotypes with the same geographic origin are clustered together, although a smaller number of genotypes with different origins are also included in the same cluster. Understanding the link between variables may be aided by the use of multivariate statistical analysis, such as PCA. These might help determine the traits nature and simplify data collection^124^. The first four principal components in this study's PCA analysis of the nine quantitative characteristics explained 88.4% of all variance, indicating a very significant link between the features under investigation (Fig. 4a). The first PC was the most important, accounting for 30% of the variance on its own. Because of their significant loadings, the features of YPP, HSW, PH, and PPP were crucial in the first PC for distinguishing the genotypes (Fig. 4c). Similar findings were found in a field pea crop by Chowdhury and Mian^125^. A method of multivariate analysis known as PCA biplot combines characteristics and genotypes in two dimensions while eliminating overlapping variations from large, complicated data sets, making it easier to identify key figures for selection (Fig. 5). As a result, PCA revealed distinct trait differentiation as well as significant variation across the five groups of the 85 field pea genotypes (Fig. 5). The traits PPP, HSW, PH, and YPP significantly contributed to describing the variations among the BFP74, BFP78, BFP76, BFP80, BFP75, BFP45, and BFP73 genotypes, and as a result, it will be possible to improve the genetic diversity of field pea genotypes through selection using these traits. Singh et al.^113^ claimed that while choosing the kind of cluster to utilize for further selection and the pattern to use for hybridization, greater focus should be placed on the characteristic that provides the greatest divergence. The correlation matrix of the observed qualities in Fig. 5 also confirmed the variability of traits determined by PCA biplot and cluster analysis.

One of the widely used statistical methods for categorizing items into groups that have a lot in common with other groups of objects is cluster analysis. The clusters will be useful for upcoming heterotic breeding since different sets of alleles may affect their traits and performance^125^. According to earlier publications by Prasad et al.^1^ and Kumar et al.^126^, great variety was present in the material under evaluation, as evidenced by the discrimination of genotyping lines into so many distinct clusters. Table 9 displays the estimated intra and inter-cluster distances for five clusters. Cluster 5 had the largest intra-cluster distance, which was followed by clusters 2, 1, and 4, whereas clusters 2 and 3 had the greatest inter-cluster distance, which was followed by clusters 1 and 3 (Table 9). In cluster 4 with 8 genotypes, we observed that variables that contribute to PPP, PL, PH, and YPP were substantially higher (Table 10). The findings of this research showed that yield-related positive and significant traits had the ability to enhance seed production. Since they demonstrated a favorable and statistically significant link with grain production, these traits were taken into account throughout the selection process. Thus, the field pea hybridization program for a variety of applications might employ these genotypes of field pea as parental sources.

In conclusions, both multivariate statistical analysis tools showed the existence of wide genetic diversity among the landraces in the study for qualitative and quantitative characteristics during the years 2022 and 2023. The results of the current research showed that there was enough genetic variation within and between genotypes, suggesting the prospect of further genetic improvement in field pea yield and traits associated with yield. The studied qualitative and quantitative traits both revealed connections between one another. The findings of the current study showed that there was significant variation in both seed yield and its related traits and resistance to powdery mildew diseases, suggesting the possibility of selecting promising gene pools that could be used as direct sources or transferred through hybridization to genotypes with high yields but disease-prone traits. The BFP77, BFP74, BFP63, BFP62, BFP43, and BFP80 genotypes were also high-yielding and resistant to PM, while check variety (BFP84) was highly resistance but lower yield; they could be selected as elite genotypes to pass to the next yield trial or for crossing purposes. BFP78, BFP72, BFP79, and BFP48 genotypes were found to be high-yielding and moderately resistant to PM. For breeding reasons, elite genotypes might be chosen from high-yielding and resistant gene pools (BFP77, BFP74, BFP63, and BFP62) and low-yielding and resistant gene pools (BFP50, BFP69, BFP53, BFP30, BFP57, and BFP65). The 85 genotypes were clearly divided into five groups of clusters using the dendrogram and heatmap-oriented cluster analysis, and there was substantial genetic variation. The genotypes BFP78, BFP77, BFP74, BFP45, BFP79, and BFP80 were identified as promising for PPP and PL in relation to yield features. All four PCs had eigen values greater than one and displayed roughly 88.4% variability. Therefore, these PCs received the attention they deserved for the following explanation. The PC1 displayed 30% of the overall variation, followed by the second and third components, which accounted for 23.9 and 18.3% of the genotype-specific total variation, respectively. While YPP, HSW, PH, and PPP were associated with PC1, DF and DM predominated in PC2. All potential pairs of clusters' genetic divergence were extremely significant. The selection of these features could be utilized as selection criteria to increase the seed yield of field pea genotypes because PH, PPP, and HSW demonstrated a substantial positive connection with YPP. The examined gene pools were extremely varied, as evidenced by the inter-cluster D^2^ value, which ranged from 2.06 to 11.89, while cluster 5 had the greatest intra-cluster distance. The mean values for PH, PPP, PL, and YPP were the highest in Cluster 4 whereas SPP had the lowest value (3.79) found in cluster 5. Maximum DM and HSW mean values were observed in clusters 5 and 3, respectively. Furthermore, it might be deduced that offspring from different crossings should exhibit a broad range of genetic diversity and had a greater capacity to identify transgressive segregants in subsequent generations. The genetic variation of the genotypes of field peas that were evaluated in this study might provide helpful information for further trait specific breeding initiatives. It is concluded that the germplasm tested in this experiment had a high degree of resistance to powdery mildew, combined with enough genetic diversity and agronomic superiority, making it suitable for use in breeding field pea varieties with both high yield and resistance to powdery mildew. This work used phenotypic selection for agronomic performance within field pea genotypes that would produce many useful field pea breeding lines for the future. In the current investigation, six genotypes BFP77, BFP74, BFP63, BFP62, BFP43, and BFP80 compared to check BFP84 were found to be highly resistant with a higher yield. These genotypes can be used to select field pea genotypes for the creation of molecular markers, mapping populations for molecular breeding, QTL identification for PM resistance, and the development of PM-resistant varieties.

### Supplementary Information

Supplementary Information.

## Data Availability

All data generated or analyzed during this study are included in this published article and its supplementary information files.
